# Advances in Slime Mould Algorithm: A Comprehensive Survey

**DOI:** 10.3390/biomimetics9010031

**Published:** 2024-01-04

**Authors:** Yuanfei Wei, Zalinda Othman, Kauthar Mohd Daud, Qifang Luo, Yongquan Zhou

**Affiliations:** 1 Faculty of Information Science and Technology, Universiti Kebangsaan Malaysia, Bangi 43600, Selangor, Malaysia; 2 Xiangsihu College, Guangxi Minzu University, Nanning 530225, China; 3 College of Artificial Intelligence, Guangxi Minzu University, Nanning 530006, China; 4 Guangxi Key Laboratories of Hybrid Computation and IC Design Analysis, Nanning 530006, China

**Keywords:** slime mould algorithm (SMA), swarm intelligence, optimization, metaheuristic algorithm

## Abstract

The slime mould algorithm (SMA) is a new swarm intelligence algorithm inspired by the oscillatory behavior of slime moulds during foraging. Numerous researchers have widely applied the SMA and its variants in various domains in the field and proved its value by conducting various literatures. In this paper, a comprehensive review of the SMA is introduced, which is based on 130 articles obtained from Google Scholar between 2022 and 2023. In this study, firstly, the SMA theory is described. Secondly, the improved SMA variants are provided and categorized according to the approach used to apply them. Finally, we also discuss the main applications domains of the SMA, such as engineering optimization, energy optimization, machine learning, network, scheduling optimization, and image segmentation. This review presents some research suggestions for researchers interested in this algorithm, such as conducting additional research on multi-objective and discrete SMAs and extending this to neural networks and extreme learning machining.

## 1. Introduction

**Figure 1:**
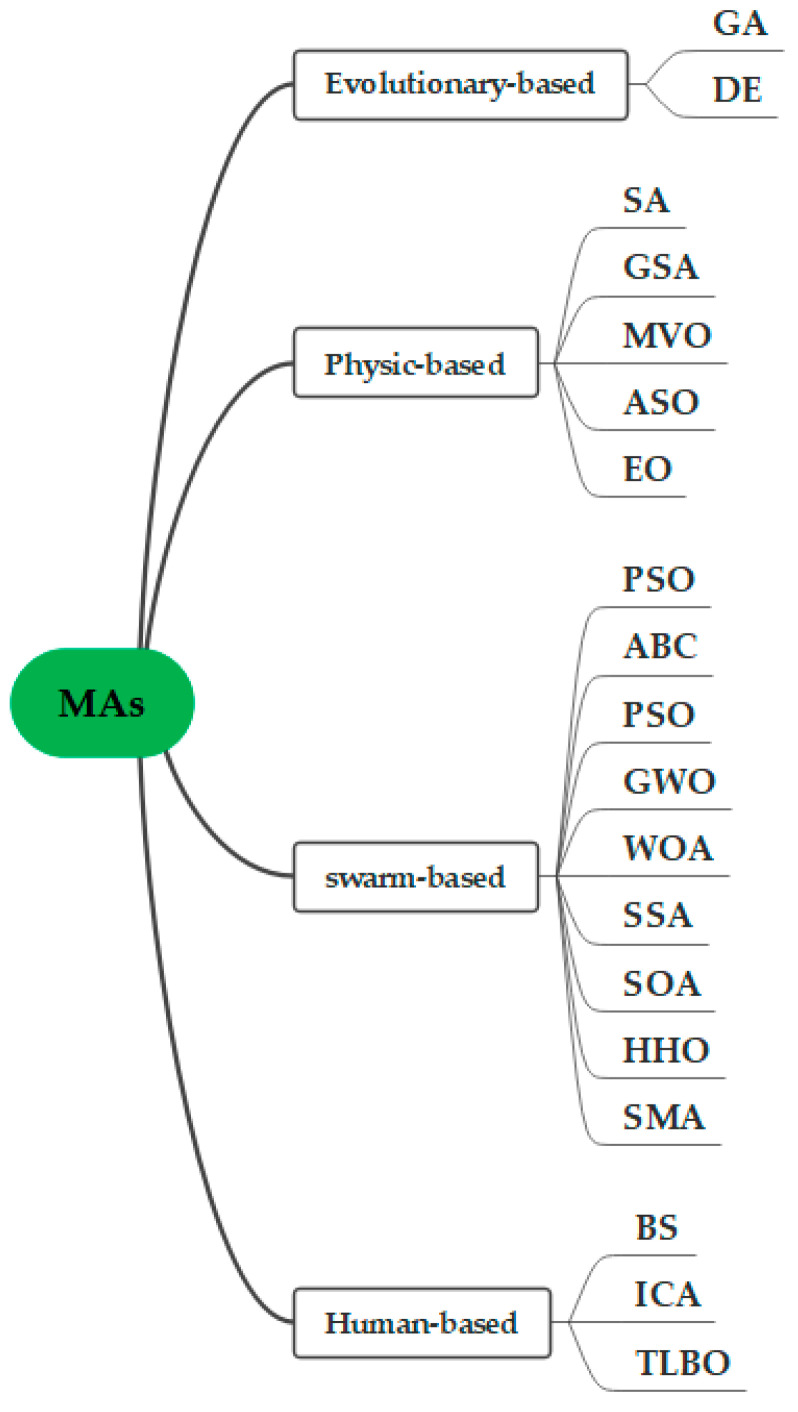
Classification of metaheuristic algorithms.

Optimization problems are commonly observed in real-life instances and many of them are NP-hard problems, which are challenging for traditional optimization methods to solve. Researchers have turned their attention to metaheuristic optimization algorithms for solving optimization problems, especially for complex, nonlinear, and high-dimensional optimization problems. In recent years, metaheuristic optimization algorithms have achieved great success in solving complex optimization problems. In general, the metaheuristic algorithms are mainly divided into evolutionary, physics-based, swarm intelligence, and human-based algorithms, as shown in Figure 1.

Evolution-based algorithms simulate Darwinian biological evolution and mainly include the genetic algorithm (GA) [1] and differential evolution (DE) [2]. Physics-based algorithms are inspired by the laws of physics and mainly include the following types: simulated annealing (SA) [3], gravitational search algorithm (GSA) [4], multiverse optimizer (MVO) [5], atom search optimization (ASO) [6], and equilibrium optimizer (EO) [7]. Swarm-based algorithms are derived from the collaborative behavior of mammals, insects, birds, fishes, and other living creatures. Representative algorithms include particle swarm optimization (PSO) [8], artificial bee colony algorithm (ABC) [9], teaching learning optimization algorithm (TLBO) [10], gray wolf optimizer (GWO) [11], whale optimization algorithm (WOA) [12], salp swarm algorithm (SSA) [13], social spider optimization (SSO) [14], seagull optimization algorithm (SOA) [15], Harris hawks optimization (HHO) [16], aquila optimizer (AO) [17], bald eagle search (BES) [18], slime mould algorithm (SMA) [19], marine predators algorithm (MPA) [20], and chameleon swarm algorithm (CSA) [21]. Finally, human-based algorithms mimic the individual/collective interactions and behaviors, social activities or lifestyles of human beings. Some of the most well-known methods presented in the literature include harmony search (HS) [22], firework algorithm (FA) [23], and the imperialist competitive algorithm (ICA) [24].

The slime mould algorithm (SMA) is a new swarm-based metaheuristic algorithm that was proposed by Li et al. in 2020, which mimics the oscillatory behavior of slime mould when locating food. Due to the simple structure and good scalability that characterize the SMA, many researchers have applied it to many fields, such as feature selection, energy, path finding, and engineering. Therefore, it is significant to introduce the recent development of the SMA and its variants in this study. The main contributions of this paper are the following:

- The mathematics model of the SMA and its pseudo-code and flowchart are analyzed;
- A statistical analysis of SMA publications is conducted;
- The various strategies and hybridizations of other algorithms to improve the model’s performance are introduced;
- Some of the recently proposed multi-objective and discrete version of SMA variants are introduced;
- The application of SMA variants is indicated;
- The advantages and disadvantages of the SMA are summarized;
- Research directions are suggested.

The sections in this study are organized and detailed as follows: Section 2 presents an overview of the research methods used in this review and the different taxonomies of research topics in the SMA; Section 3 presents the SMA’s concept and the SMA’s mathematic model, its pseudo-code and flowchart; Section 4 introduces the details concerning SMA’s variants and the improvements; Section 5 presents the wide range application domain of the SMA and identifies its variants; Section 6 discusses the advantages and disadvantages of the SMA; Section 7 presents the conclusions and some potential future research directions.

## 2. Research Methodology and SMA Survey Taxonomy

### 2.1. Research Methodology

**Figure 2:**
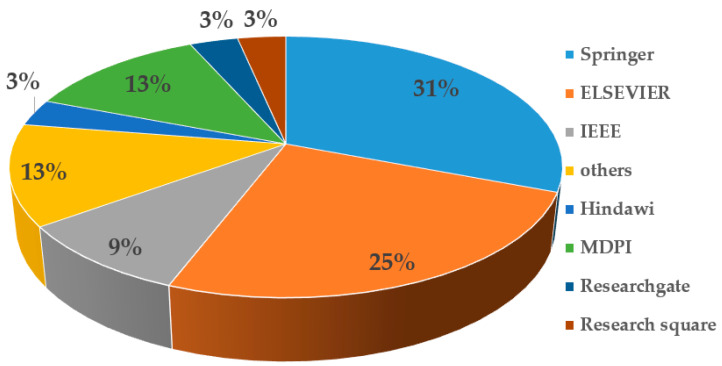
The number of publications based on publishers since 2022.

In this paper, we propose a review of the SMA with its variants and application domains. To collect the papers related to the SMA, we conducted an extensive search in Google Scholar using the key words of “Slime Mould Algorithm” or “SMA” appearing since 2022. A total number of 130 papers were obtained from various well-known publishers, such as Springer, Elsevier, IEEE, Hindawi, MDPI, Taylor & Francis, Research square, and ResearchGate. The statistics of the review are indicated in Figure 2. Table 1 lists 3 countries ranked according the number of publications on SMAs since 2022. 

### 2.2. SMA Survey Taxonomy

This paper reviewed 130 publications on SMAs since 2022. For these 130 papers, we summarized and analyzed them from two perspectives. Firstly, due to the existence of the extensive research conducted on a single objective, we divided the publications into two categories depending on the improved approach: the strategy adding and hybridization methods. Compared with single-objective and continuous versions in the field, the number of multi-objective versions and discrete version publications are relatively lower. Therefore, we separately classified these publications into multi-objective and discrete SMAs. Secondly, we analyzed and summarized the 130 papers obtained from the perspective of application domains, indicating the research trends of SMAs in different application domains. Based on the content of the 130 papers, we identified 7 categories: engineering optimization, energy optimization, machine learning, image segmentation, network, scheduling optimization, and others. Engineering optimization focuses on modeling and optimizing solutions for various industrial manufacturing, engineering design, and parameter optimization problems. Energy optimization includes building various models for different energy problems and using the SMA to optimize the solutions. Machine learning represents the combination of SMA and various machine learning methods, such as SVM, to perform optimization tasks such as feature extractions, classifications, and predictions. Image segmentation is used to improve the factors of segmentation accuracy and quality by combining SMAs and image segmentation models. Scheduling optimization uses the SMA to solve scheduling optimization problems. Others represent the use of the SMA to optimize only the benchmark numerical functions. 

## 3. Slime Mould Algorithm (SMA)

### 3.1. Concept of SMA

**Figure 3:**
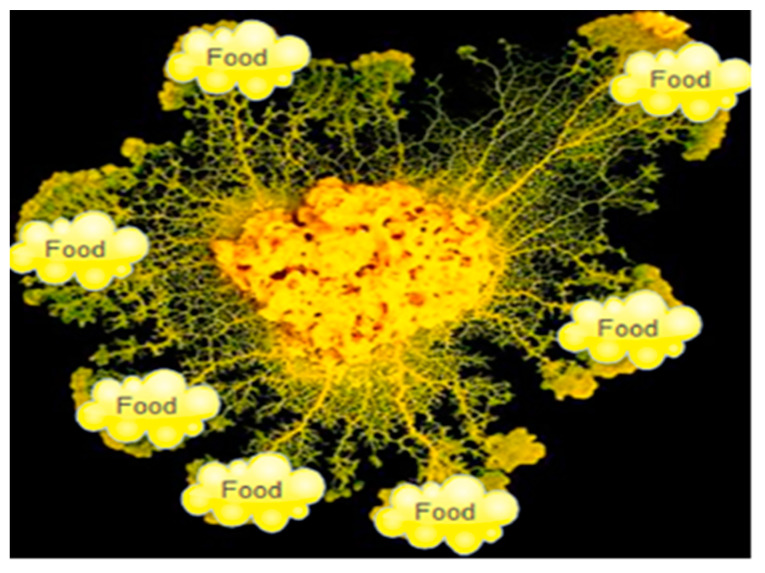
Foraging morphology of slime mould [25].

The slime mould algorithm (SMA) was proposed based on the mathematical simulation of the oscillatory activity of slime mould during foraging [25]. Slime mould has the properties of oscillating and contracting when it finds food. The foraging behaviors of slime moulds are classified into three areas: finding food, approaching the food, and digesting the food by enzymes. In the process of migration, the front end of the slime mould disperses into a fan-like shape and forms a network of veins with different thicknesses across multiple food sources, as shown in Figure 3.

The network’s thickness is, to some extent, proportional to the size of the food source. When the vein targets a high-quality food source, a biological oscillator generates a stronger propagating wave that increases the flow of cytoplasm, thus creating a thicker network. Therefore, the optimal path from the slime mould to the food source is established in this positive and negative feedback mechanism.

Slime mould is also likely to search for other types of food sources. When the information obtained is inadequate and incomplete, following the heuristic or empirical rule that is based on insufficient information would be the best way for slime mould to evaluate its current position and determine whether to move. The possibility of leaving its location is decreased when the slime mould finds a better source of food [26]. However, slime mould still makes good use of its various food sources at the same time because of its unique biological characteristics. Even if a better food source is found, slime mould allocates parts of its biomass to exploit both food sources simultaneously [27].

Slime mould also adjusts its search pattern dynamically based on the provenance of the food. When high-quality food is attainable, slime mould would focus its search on the food that is available using a region-limited search method [28]. If the original food deteriorates to a low concentration, slime mould abandons it to explore an alternative high-quality food source [29].

### 3.2. Mathematical Model of the SMA

The mathematical model used to update the slime mould’s position is represented as Equation (1):

(1) $$\vec{X(t+1)}=\left\{\begin{array}{cc} rand\cdot (UB-LB)+LB & rand<z\\ \vec{X_{b}(t)}+\vec{vb}\cdot \left(\vec{W}\cdot \vec{X_{A}(t)}-\vec{X_{B}(t)}\right) & r<p\\ \vec{vc}\cdot \vec{X(t)} & r\geq p \end{array}\right.$$

where LB and UB are the lower and upper boundaries of the search scope; rand and r are random values obtained within the range of [0, 1]; z=0.03 is a parameter; $\vec{X_{b}(t)}$ indicates the position of the strongest food odor present at that moment; $\vec{vb}$ is a parameter with values within [−a,a]; $\vec{vc}$ is also a parameter fluctuating linearly from 1 to 0 with the number of iterations t; $\vec{W}$ represents the slime mould’s weight determined by Equation (4); $\vec{X_{A}(t)}$ and $\vec{X_{B}(t)}$ are two randomly selected agents’ positions in the population; and $\vec{X(t)}$ indicates the current position of the slime mould.

The value of p is calculated using Equation (2):

(2) p=tanh⁡|S(i)−DF|

where i∈1,2,...,n, S(i) denotes the fitness $\vec{X}$, and DF represents the best fitness obtained to date.

The value of a to determine the boundary of $\vec{vb}$ is defined using Equation (3):

(3) $$a=\arctan h\left(1-\frac{t}{\max\_t}\right)$$

where max_⁡t indicates the maximum number of iterations.

The slime mould’s weight $\vec{W}$ is calculated using Equation (4):

(4) $$\overset{⃑}{W}(SmellIndex(i))=\left\{\begin{array}{cc} 1+r\cdot \log\left(\frac{bF-S(i)}{bF-wF}+1\right) & condition\\ 1-r\cdot \log\left(\frac{bF-S(i)}{bF-wF}+1\right) & others \end{array}\right.$$

(5) $$\vec{SmellIndex}=sort(\vec{S})$$

where condition represents how S(i) is ranked in the first half of the population; r represents a random number in [0, 1]; bF represents the optimal fitness level obtained in the current iteration; wF is the worst fitness level obtained in the current iteration; and $\vec{SmellIndex}$ denotes the result of the ascending order of fitness values (in the minimization problem).

### 3.3. The Pseudo-Code and Flow Chart of the SMA

**Figure 4:**
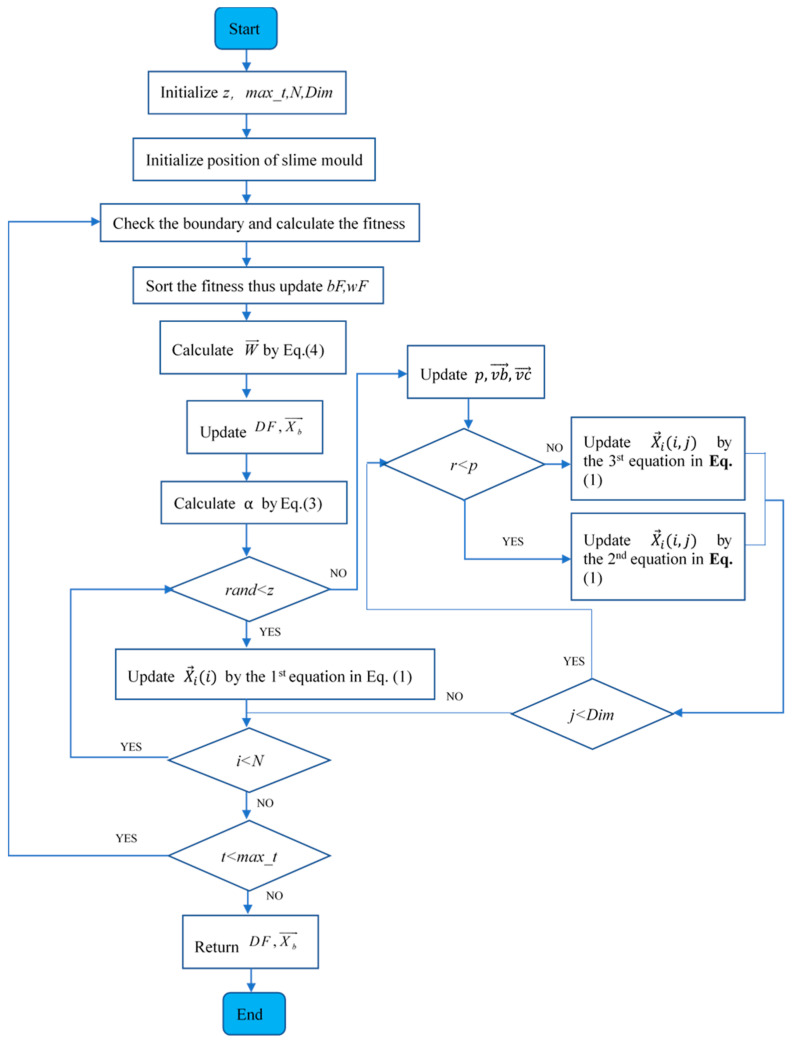
Flowchart of SMA.

The pseudo-code and flow chart of the SMA are presented in Algorithm 1 and Figure 4.
**Algorithm 1:** Pseudo-code of SMA1. Initialize the parameters z,max_⁡t,N,Dim;2. Initialize slime mould’s random location $\vec{X_{i}}(i=1,2,\cdots ,N)$;3. **While** (t≤max_⁡t)4.  Check the boundary and determine the fitness $\vec{S}$;5.  Sort the fitness $\vec{S}$;6.  Update $bF,wF,DF,\vec{X_{b}}$;7.  Calculate $\vec{W}$ as per **Equation** (4);8.  Update $p,\vec{vb},\vec{vc},A,B$;9.  **For** each search agents10.   Update location as per **Equation** (1);11.  **End For**12.  t=t+1;13. **End While**14. **Return** $DF,\vec{X_{b}}$;

## 4. Recent Variants of the Slime Mould Algorithm

**Figure 5:**
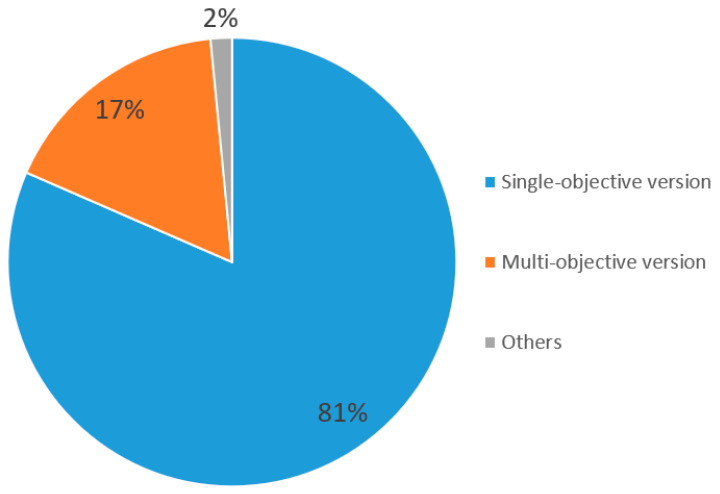
Distribution of SMA publications based on the single/multi-objective since 2022.

**Figure 6:**
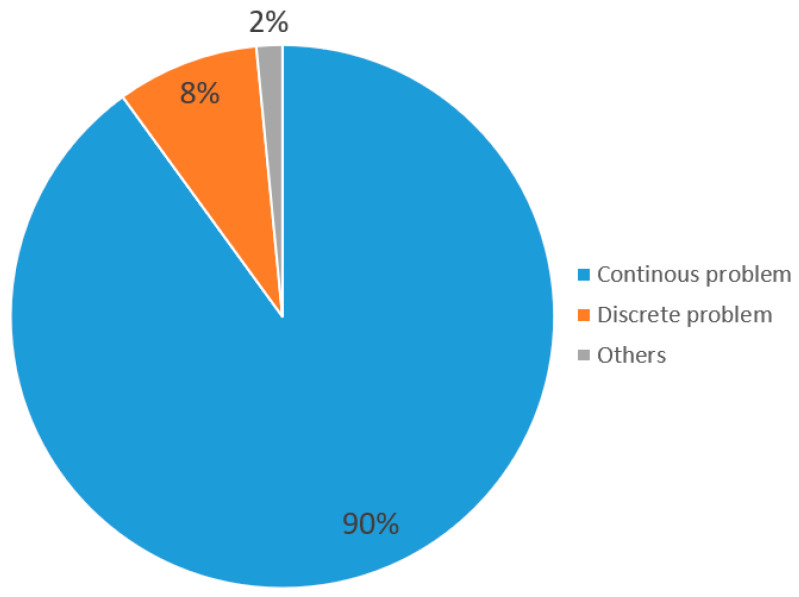
Distribution of SMA publications based on continuous/discrete problems since 2022.

The basic SMA was mainly used in research for solving continuous single-objective optimization problems when it was first proposed in 2020. The SMA has been widely used by researchers in the field and numerous SMA variants have presented improved techniques, such as strategy adding and hybridization with other algorithms for solving different kinds of problems (e.g., single/multi-objective and continuous/discreate). According to 130 papers on SMAs collected from well-known publishers between 2022 and 2023, most of these SMA variants were applied to single and continuous problems as indicated in Figure 5 and Figure 6. To the best of our knowledge, the classification of SMA variants in the literature is not clear and most studies classify them based on their methods and the problems they face and resolve. As shown in Figure 5 and Figure 6, 81% of the reviewed papers belong to the single-objective category and 90% belong to the category of continuous problems. Therefore, most of the SMA variants were used to solve single-objective and continuous optimization problems and it was not necessary to categorize them. Therefore, we classified these 130 papers into the following four categories: modified, hybridized, discrete, and multi-objective versions. 

### 4.1. Modified Version of the SMA

Modified versions of SMA variants [30] refer to those that employ the methods of simplifying, removing, replacing original operators, or adding new operators, while the framework remains unchanged. We can adopt the SSMA [31] proposed by Yuanye Wei et al. as an example. In the SSMA, the third equation in Equation (1) was removed and the original oscillation factor was replaced by cosine function to form a simple version of SMA variants. We refer to those methods mentioned above as strategy adding, which are often adopted by researchers in the field. The relevant SMA variants adding different strategies are presented in Appendix A. 

#### 4.1.1. Opposition-Based Learning (OBL)

As shown in Appendix A, opposition-based learning was the most popular strategy that the researchers used for the SMA variants. OBL was first proposed by Tizhoosh HR in 2005 [32] and aimed at increasing the populational multiformity to jump out local optima.

In the studies of [33,34,35,36,37,38,39,40], the researchers used basic OBL techniques to improve their exploration of SMA variants.

Izci D et al. [33] improved the exploration of the original SMA with a modified OBL in their study. Dipak Kumar Patra et al. [34] developed an enhanced SMA by incorporating the quasi opposition-based learning (QOBL) mechanism in their study. Krishna Gopal Dhal et al. [35] proposed an improved SMA (called ISMA) based on a random OBL and DE’s mutation strategy. Liang Xu et al. [36] presented an ISMA by adding an improved random OBL to increase convergence ability in the late iteration and prevent local optimizations from occurring. Sengathir J. et al. [37] utilized the OBL strategy in the proposed AOLISMA for self-adjusting for exploration purposes. In addition, several modified versions of OBL exist in the literature, namely, adaptive OBL, quasi opposition optimization learning [38], and random opposition-based learning [39], which further increase population diversity and improve populations’ capacities to avoid local optimality. Houssein EH et al. [40] introduced a modified OBL that utilized-Lévy’s flight distribution. 

#### 4.1.2. Chaotic Strategy

The chaotic strategy adopts the second position after OBL in Appendix A. Chaos, as a non-linear system commonly observed in nature, is applied by many researchers to optimize search problems due to its random, traversal, and regular nature [41]. The researchers added the chaotic strategy to the SMA for maintaining the population diversity, thus avoiding the occurrence of local stagnation. 

Rizk-Allah RM et al. [42] proposed a version of CO-SMA by adding a chaotic strategy. The chaotic search strategy can enhance the neighborhood search around the best position. Li YiFei et al. [43] embedded polynomial chaos expansion (PCE) into the SMA for multi-parameter identification of concrete dams. Yin Shihong et al. [44] organized a set of chaotic sequences using a chaotic grouping mechanism (CGM) to improve the population diversity results. Xuebing Cai et al. [45] proposed a multi-objective SMA, called CRFSMA, which incorporated a chaotic mechanism to increase its search ability. Yuan L et al. [46] presented an enhanced algorithm of the SMA, called ECSMA, where elite and chaotic stochastic strategies were embedded into the structure to maintain a good balance between exploration and exploitation behaviors. Dhawale D et al. [47] used a chaotic SMA (named CSMA) combined with a tent chaotic function. Chen H et al. [48] replaced the original random initialization of the SMA with chaotic maps in order to enhance the diversity of the population. Zhong C et al. [49] employed an adaptive chaos control method to solve reliability-based design optimization (RBDO) problems. Miao H C et al. [50] presented an MSMA that adopted the elite chaotic search strategy (ECSS) to enhance explorations conducted near elite individuals. Bhadoria A et al. [51] used a singer map chaotic search strategy to improve a local search during the exploitation phase. Sarhan S et al. [52] suggested an ESMO incorporating a chaotic strategy and an elitist group. Singh T et al. [53] proposed a chaotic slime mould algorithm (CSMA) with the integration of chaotic sequences during the optimization process.

#### 4.1.3. Mutation and Crossover Operators

The concepts of mutation and crossover are derived from the genetic algorithm and differential evolution. Both algorithms conduct the processes of mutation, crossover, and selection to generate the next generation of the population. Researchers have used mutation and crossover operators to increase populational diversity, thus improving exploration and exploitation outcomes.

Some researchers have used both of the mutation and crossover operators as multi-strategies for SMAs. Ramin Ghiasi et al. [54] embedded two mutation and crossover operators to a binary slime mould algorithm (ABSMA) to overcome stagnation. Deng L et al. [55] presented an MSMA in which a mutation operator was added to generate a new search equation to maintain the balance of exploitation and exploration, and an adaptive mutation probability was constructed to avoid premature convergence and maintain the population diversity. Qiu F et al. [56] proposed an improved algorithm (ISMA) by combining two strategies of Cauchy and crossover mutations based on the DE, to promote the coordination of global exploration and local exploitation.

Various SMA variants use mutation and crossover operators. Many versions of SMA variants with added mutation mechanisms have been proposed and have proved mutation as a useful method. Lin H et al. [57] developed an ASMA where a trigonometric-based mutation operator and a double-based best mutation operator were used to improve the convergence speed, and a binomial crossover operator was also used to increase the population diversity. Rizk-Allah RM et al. [42] proposed a modified SMA with a chaotic search strategy (CSS) and crossover opposition strategy (COS) added to avoid trapping in local optima. Yin S et al. [58] added the random difference mutation strategy to the proposed EOSMA to help the algorithms jump out from the local stagnation. Pawani et al. [59] introduced a wavelet mutation into an SMA to avoid local optimal outcomes. Zheng R et al. [60] integrated multiple mutation operators and the restart method into an SMA. Yang H et al. [61] introduced a mutation mechanism and dynamic weight coefficient to the proposed SMA to solve problems of slow convergence and low optimization precision results. Yang P et al. [62] added a mutation operation to the SMA’s position update to improve the global optimization ability of the SMA. The crossover operator adding method has also presented its advantages in many SMA variants. Qi A et al. [63] presented a novel SMA variant, named SDSMA. In the SDSMA, a directional crossover mechanism was added to enhance the balance of exploration and exploitation, thus helping the SDSMA to increase the convergence speed and accuracy. Ma TX et al. [64] introduced an improved crossover operator into the improved artificial bee colony–slime mould algorithm to improve the convergence speed.

#### 4.1.4. Lévy Flight

Lévy flight is a probability distribution proposed by Paul Pierre Lévy [65], which specially enhances the global search capacity of an SMA to prevent it from being stuck in the local optimal position. The Lévy flight mechanism combines short-distance walking with long-distance jumping routes to search for space. Therefore, the researchers used it to improve the global search ability of the algorithm. 

Ling Zheng et al. [66] proposed the Lévy flight-rotation SMA (LRSMA), which utilized a variable neighborhood Lévy flight. He W et al. [67] used a Lévy flight sequence to increase the convergence speed of the SMA. Pan JS et al. [68] proposed an MFSMA, which was added to an adaptive Lévy flight. Qi A et al. [63] presented an SDSMA combining adaptive Lévy diversity and directional crossover mechanisms. Qiu F et al. [69] used Lévy flight to help the SMA jump out the local optimum position. Jui JJ et al. [70] integrated the Lévy distribution into an SMA for solving the local optima problem. Kundu T et al. [71] introduced Lévy flight into an SMA to improve the global search ability.

#### 4.1.5. Elite Strategy

The elite strategy is widely adopted by researchers for many SMA variants. The concept of the elite strategy is to introduce elite individuals, which are used to generating the current solution corresponding to the elite solution. Then, elite solutions are compared to the current solutions so that the best individuals are chosen as the next generation. Therefore, the elite strategy not only increases the search scope of an algorithm, but also improves the diversity of the population.

Yuan L et al. [46] proposed an enhanced SMA called ECSMA, where the elite strategy was embedded into the structure. Miao H C et al. [50] added an elite chaotic search strategy (ECSS) to the proposed MSMA to enhance the exploration around elite individuals. Sarhan S et al. [52] incorporated an elitist group mechanism in the ESMO. Kaveh A et al. [72] adopted an elitist strategy in the replacement phase of an SMA to increase the convergence rate of the proposed ISMA. Luo Qifang et al. [73] proposed an MOEOSMA by adding an elite archiving mechanism to promote the overall convergence speed.

#### 4.1.6. Greedy Selection (GS)

The greedy selection strategy is one of the most widely used strategies adopted by researchers for SMA variants. Liu J et al. [74] proposed a multi-strategy information interaction and optimally oriented initialization (MSII-SMA) in their study. The greedy selection strategy was employed for building the information exchange model to provide a better solution for the subsequent iteration. Shubiao Wu et al. [75] adopted a greedy selection strategy to increase the convergence rate of the proposed GBSMA. Yin S et al. [76] updated the individual and global historical optimal values with the greedy strategy, resulting in the acceleration of the convergence.

#### 4.1.7. Fuzzy

The fuzzy strategy is commonly adopted for SMA variants. Prabhu M et al. [77] proposed a fuzzy-based slime mould optimization for the carrier frequency offset (FSM-CFO). The fuzzy rules were designed and applied for the assignment of resource units (Rus) to a certain job. Al-Kaabi M et al. [78] presented a multi-objective slime mould algorithm (MOSMS), where the fuzzy set theory was used to obtain an optimal solution. Yutong G et al. [79] presented a fuzzy SMA (named FSMA), where the control parameters were updated by a fuzzy system.

#### 4.1.8. Neighborhood Search (NS)

The neighborhood search (NS) is a useful strategy used to enhance the exploitation ability of algorithms. Yuanfei Wei et al. [80] added a variable NS strategy in the proposed EOSMA to increase the exploitation outcomes. Zhou X et al. [81] introduced an all-dimensional neighborhood search strategy for an SMA (LASMA) to explore the search space more effectively.

#### 4.1.9. Others 

In addition to the strategies previously mentioned in this study, there are numerous additional strategies that researchers usually add to SMAs, including the Nelder–Mead simplex search [33], bee-foraging learning operator [39], dispersed foraging strategy [40],orthogonal learning [82], dynamic random search [55], sigmoid function [67,83], and Gaussian strategy [75,84]. These strategies have improved the performance of SMAs and are important directions for researchers.

### 4.2. Hybridized Version of the SMA

The hybridization of the SMA with other algorithms is an important method that researchers use to improve the performance of SMAs. Because SMAs have a strong scalability factor, it is natural for SMAs to hybridize with other algorithms. Appendix B shows the relevant SMA variants hybridized with other algorithms based on the 130 publications that were reviewed.

When conducting a literature review of these 130 papers, we concluded that two approaches were generally used by researchers to perform algorithm hybridizations: framework modification and operator fusion.

The framework modification approach modifies the original SMA by adding the other algorithm’s strategy or combining both algorithms to solve the same optimization problem. For example, A. A. Ewees [85] combined the updating technique of the firefly algorithm (FA) with the structure of the SMA. The operators of the SMA and FA competed to update the solution, which maintained the balance between the exploration and exploitation factors during the search process. This approach allowed the algorithms to operate independently; however, they could exchange and modify inter-population information, taking advantage of multiple algorithms to complete the solution [86].

The operator fusion replaces or modifies the SMA operators with other algorithms or combines different operators from both algorithms to solve the problems of the original SMA. The operator fusion approach is mostly apparent in the hybrid version of SMA combined with the EO, EO, and GA. As for the hybridization of SMA and EQ in [58,73,76,80], the operators observed that the best SMA was replaced by the first solution in the equilibrium pool of EO. Additionally, the mutation and crossover operators of EO and GA are usually adopted to update the position during the search for exiting the local optimum position.

Compared with both approaches, the operator fusion was more popular than the framework modification because it was relatively easy to modify and replace the SMA original operator without changing the framework and structure, resulting in a reduced computational time. However, achieving a complete understanding of both algorithms’ mathematical models is a premise for the implementation of the operator fusion approach and a challenging task for the researchers.

#### 4.2.1. Hybridization with the Equilibrium Optimizer (EO)

Yin S et al. [58,76] replaced the original search operator of an SMA with a concentration update operator of EO to improve the search efficiency of the algorithm. Yuanfei Wei et al. [80] changed the random search operator in an SMA with an EO strategy to enhance the population diversity. Luo Qifang et al. [73] integrated the equilibrium pool’s strategy to the proposed EOSMA at the stage of population initialization, helping the proposed algorithm to improve the exploration outcomes.

#### 4.2.2. Hybridization with the Differential Evolution (DE)

Chen H et al. [48] proposed a CHDESMA which was added with the crossover and selection operators derived from DE to assist the algorithm with the existing, local optimal position. Shubiao Wu et al. [75] proposed a GBSMA combined with DE’s position updating mechanism to enhance the exploration capability. 

#### 4.2.3. Hybridization with the Support Vector Machine (SVM)

Yuheng Guo et al. [87] employed the SMA approach to optimize the parameters of an SVM for an ancient glass classification. Gao H et al. [88] proposed a prediction model on the basis of a modified SMA and SVM algorithm. Javidan SM et al. [89] combined an SMA with an SVM classifier to diagnose apple tree diseases. Shi B et al. [90] combined the proposed JASMA with a common kernel learning SVM to conduct an analysis of recurrent spontaneous abortion (RSA). 

#### 4.2.4. Hybridization with the Whale Optimization Algorithm (WOA)

Anji Reddy Vaka et al. [91] proposed a hybrid SMA with WOA, where the WOA technique performed an exploration in the first half of the iterations and the SMA method performed an exploitation in the second half of the iterations. Bhandakkar AA et al. [92] improved the searching behavior of an SMA by incorporating it with WOA. Li X et al. [93] combined an SMA and an WOA with two fusion measures to enhance the global optimization ability. They replaced one parameter of the WOA with a parameter of the SMA and introduced the position updating strategy of the WOA into each slime mould individual.

#### 4.2.5. Hybridization with the Simulated Annealing (SA) Algorithm

Izci D et al. [30] proposed an opposition-based hybrid SMA with an SA algorithm to improve the exploitation and exploration factors of the original SMA. Leela Kumari Ch et al. [94] used an SA algorithm to help the SMA avoid trapping the local optimum.

#### 4.2.6. Hybridization with the Teaching–Learning-Based Optimization (TLBO)

Zhong C et al. [49] suggested a hybrid SMA with TLBO, which enhanced the convergence speed of the SMA. Kundu T et al. [71] utilized the characters of the SMA and TLBO to maintain a good balance between the exploitation and exploration factors.

#### 4.2.7. Hybridization with the Seagull Optimization Algorithm (SOA)

Bhadoria A et al. [51] enhanced the exploration and exploitation capabilities of an SMA by sequentially hybridizing an SMA and SOA. Das G et al. [95] hybridized an SMA and SOA to improve the global and local search spaces.

#### 4.2.8. Hybridization with the Artificial Bee Colony (ABC)

Ma TX et al. [64] introduced the artificial bee colony (ABC) algorithm to hybridize an SMA to improve its search ability and avoid local minima. Chen X et al. [96] added the position update mechanism of the artificial bee colony into the SMA to jump out of the local opima in the process of image segmentation. 

#### 4.2.9. Others 

Moreover, researchers have hybridized an SMA with a sine cosine algorithm [83], marine predators algorithm [85], particle swarm optimization [97], evolutionary algorithm [98], firefly algorithm [99], gray wolf optimization algorithm [100], gradient-based optimizer [101], quadratic approximation [102], tournament selection [103], artificial neural network [104], moth-flame optimization algorithm [105], pattern search algorithm [106], and support vector regression [107]. These hybrid SMA variants indicated their benefits, such as the good balance between exploration and exploitation capabilities, good convergence speed, ability to avoid premature convergence, and reduced computation time. 

### 4.3. Multi-Objective Version of an SMA

Multi-objective problems are an important branch of the research conducted on optimization problems. The general mathematical model of the multi-objective optimization is presented in Equation (6):

(6) $$minmize\;\;y=f\left(x\right)=\left(f_{1}\left(x\right),f_{1}\left(x\right),\cdots ,f_{k}\left(x\right)\right)\;subject\;to\;\;e(x)=\left(e_{1}\left(x\right),e_{1}\left(x\right),\cdots ,e_{m}\left(x\right)\right)\leq 0\;\mathrm{where}\;x=(x_{1},x_{2},\cdots ,x_{n})\in X\;y=(y_{1},y_{2},\cdots ,y_{n})\in Y$$

From Equation (6), we can observe that the multi-objective optimization has many functions, and some of them are in conflict and compete with each other. The optimization of one function can be achieved at the expense of others. It is impossible to attain the best solution for all functions. The best solution can be attained from the coordination and compromise of various functions. The multi-objective optimization is a challenging task for SMAs.

Compared with a single-objective version of an SMA, the multi-objective version of an SMA obtained 17% of the reviewed publications between 2022 and 2023 (see Figure 5). Therefore, the multi-objective version of the SMA requires more attention from researchers. Son, P. V. H. et al. [108] proposed an AOSMA to find solutions for a construction project’s multi-objective optimization problem. Yin Shihong et al. [44] presented a multi-objective SMA (IBMSMA) and applied it to the multi-objective truss optimization problem. Cai Xuebing et al. [45] also proposed an MOSMA, and the simulation test demonstrated that the MOSMA attained the best convergence, accuracy, and diversity results among the compared multi-objective algorithms. Yin S et al. [76] applied a proposed multi-objective EOSMA (MOEOSMA) to the inverse kinematics of manipulators. Al-Kaabi M et al. [78] presented an MOSMA for attacking multi-objective optimal power-flow problems. Luo Qifang et al. [73] proposed an MOEOSMA and assessed it for engineering problems, which indicated its overall efficiency. Peng C et al. [107] proposed an MOSMA and optimized SVR parameters by MOSMA for global convergence. Sadasiva Behera et al. [109] used an MOISMA to solve a multi-renewable source-based energy management problem and this MOISMA-based energy management outperformed the other models. Houssein EH et al. [110] proposed an MOSMA that proved its superiority to Pareto set proximities in an experiment. Yacoubi S et al. [111] introduced an MOOSMA for numerical association rule mining (NARM). Zhang Y et al. [112] proposed a process parameter optimization method for laser cladding combined with MOSMA and a support vector regression model. Son PV et al. [113] employed a multi-objective version of SMA (ASSMA) to improve the SMA’s performance via the Pareto front. When comparing the results, it can be observed that the ASSMA model shows good diversification outcomes. 

### 4.4. Discrete Version of the SMA

A discrete problem is an important domain of the optimization problem, such as the 0-1knasack problem, traveling salesman problem (TSP), and job shop scheduling problem (JSSP). In this section, the discrete version of an SMA refers to SMA variants that researchers use as discrete mechanisms to discretize the original continuous SMA in order to solve discrete problems. In contrast to the continuous version of SMA, the discrete version of SMA considers only 8% of publications reviewed between 2022 and 2023, as shown in Figure 6. Therefore, it is an important direction for further development of SMA variants.

Most researchers use discrete original SMAs by encoding the continuous valve of a solution into a binary value without changing the framework of the original SMA. Hu J et al. [40] proposed a binary-dispersed foraging SMA (BDFSMA), which was promising in terms of feature selection. Ghiasi Ramin et al. [54] introduced a binary SMA (ABSMA) to better classify the structural damage that occurred. Feng Qiu et al. [56] converted an SMA into a binary version with a transfer function and used it for gene selection purposes. Feng Qiu et al. [69] also mapped the proposed GLSMA to a binary space via the transformation function and applied for the feature selection. Yuanfei Wei et al. [80] made the proposed EOSMA discrete with a sort-order-index (SOI)-based coding for a job shop scheduling problem (JSSP). Zhou X et al. [81] used a V-shaped transfer function to convert a proposed LASMA into a binary algorithm. The experiments showed that bLASMA produced better results regarding the convergence speed and accuracy concerning optimization problems and feature selection parameters. H.S Hassan et al. [114] proposed a BSMA, which converted continuous space into discrete space using the transformation function. Rifat Md Sayed Hasan et al. [115] employed a binary SMA to solve the unit commitment problem (UCP). Dan Li et al. [116] used the double-stranded chromosome encoding method and the solution space bisection decoding method to compose a GCSMA model and applied it to the flexible job shop scheduling problem (FJSP). MS K [117] proposed a discrete time-based slime mould optimization providing effective support to the buck converter-based MPPT controller for SPV systems. 

## 5. Applications of the SMA 

**Figure 7:**
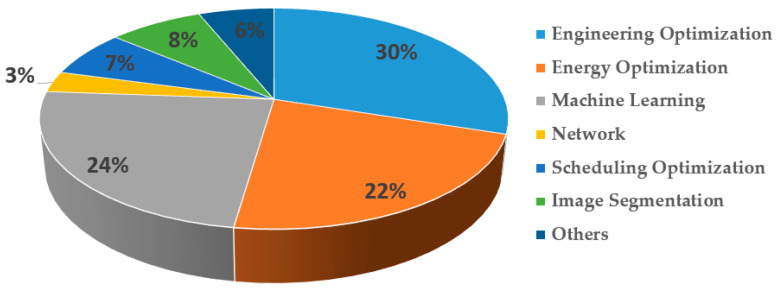
Distribution of publications based on their application domains since 2022.

The SMA and its variants have been applied to various fields of application, that include the energy, agricultural, forestry, medical and health, IT, manufacturing, electronics and communication, education, and financial industries. In this section, we classified the 130 reviewed publications into seven categories: engineering optimization, energy optimization, machine learning, image segmentation, network, scheduling optimization, and others, as shown in Figure 7. 

### 5.1. Engineering Optimization

**Figure 8:**
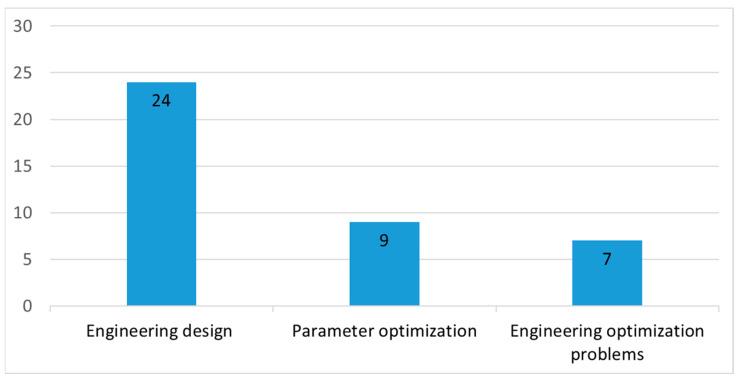
Detailed classifications and numbers of the reviewed literature for engineering optimizations.

Engineering optimization is in first position in the application domains, as shown in Figure 7. We divided engineering optimization into three categories: engineering design, engineering optimization problems, and parameter optimization, as shown in Figure 8.

#### 5.1.1. Engineering Design

Optimizing real-life engineering design problems is challenging and many applications must address the NP-hard problems. The real-world engineering design optimization problem is the achievement of maximum or minimum objections under some predefined constraints. Usually, a number of design constraints, such as dimensions, strength, and rigidity, are nonlinear and complex, which lead to difficulties in finding a solution. Because the SMA has the advantages of its simple structure and strong exploration ability, researchers have developed various SMAs for solving engineering design problems.

As an SMA was firstly proposed to solve single-objective application problems, SMA variants were mostly employed for solving single-objective engineering design problems. Houssein EH et al. [82] evaluated the proposed SMA in three engineering design problems. In the literature, many researchers have focused on structure optimization problems. Yin Shihong et al. [44] employed the proposed IBMSMA and evaluated it in a multi-objective truss. Li Yuan et al. [46] applied the presented ECSMA to four structural design issues of welded-beam, PV, I-beam, and cantilever beam design problems, achieving excellent results. Chen H et al. [48] assessed the presented CHDESMA for four real-world engineering problems, which indicated that CHDESMA presented a competitive performance. Deng L et al. [55] evaluated the proposed MSMA using several practical engineering issues, which proved that the proposed algorithm was more efficient and robust than other algorithms. Yin S et al. [58] applied the proposed EOSMA to nine engineering design problems. Zheng R et al. [60] employed three typical engineering design problems to verify the proposed ESMA’s performance. Qi A et al. [63] proposed a SDSMA and applied it to three real-world engineering design problems. Jui JJ et al. [70] evaluated the proposed LSMA in 23 well-known benchmark test functions and the welded-beam design problem. Kundu T et al. [71] evaluated the proposed LSMA-TLBO in six engineering design problems. Liu J et al. [74] compared the proposed MSII-SMA with other algorithms for engineering design optimization problems and the experimental results demonstrated the universality, reliability, and preponderance of MSII-SMA in dealing with engineering design constraint optimization problems. SMA was also developed further for multi-objective engineering design problems. Shubiao Wu et al. [75] proposed a GBSMA for solving truss structure optimization problems. Kaveh A et al. [72] used the proposed ISMA for three large-scale benchmark dome trusses. Qifang Luo et al. [73] used the proposed MOEOSMA to solve eight real-world multi-objective constraint engineering problems. Qifang Luo et al. [73] used the proposed MOEOSMA to solve large-scale truss structure optimization problems. Örnek BN et al. [83] tested the performance of the proposed SMA on the designs’ optimizations and the results demonstrated the proposed SMA had a better ability to jump out of local optima. Leela Kumari Ch et al. [94] presented a hybrid version of an SMA and assessed it in 11 kinds of interdisciplinary engineering designs. In contrast to the typical engineering design applications described above, few SMA variants are applied to perform reliability-based design optimizations (RBDO). Das G et al. [95] used the proposed TLSMA for solving five reliability-based design optimization problems in their study.

Chauhan S et al. [99] tested the proposed PSEASMA and SSEASMA in classical engineering design problems to prove their superiority over other algorithms. Bala Krishna A et al. [106] proposed a hybrid SMA variant called hSMA-PS and applied it to nine classical engineering problems. Houssein EH et al. [110] used an MOSMA for the real-world multi-objective optimization of a helical coil spring and four multi-objective engineering design problems.

Yin S et al. [118] verified the performance of a proposed DTSMA on nine engineering design problems. Lingyun Deng et al. [119] presented an AGSMA for solving practical engineering problems, which demonstrated the AGSMA’s superiority over the other algorithms to which it was compared.

#### 5.1.2. Engineering Optimization Problem

The engineering optimization problem has always been a hot research topic and SMA has been utilized in numerous applications, such as the economics, combinational optimization, and energy sectors. Rizk-Allah RM et al. [42] employed the CO-SMA to optimize wind turbine energy costs. Shahenda Sarhan et al. [52] suggested a proposed ESMO for handling optimal power flow (OPF). Wenhe He et al. [67] proposed an unresolved peaks analysis algorithm, which was based on the sigmoidal membership function, Lévy flight, and slime mould algorithm (SLSMA), for microchip electrophoresis (ME) signal detection. Chakraborty P et al. [102] proposed a hybrid SMA for three engineering optimization problems. From the evaluations, it was observed that the HSMA was an efficient algorithm for real-life problems. Rifat Md Sayed Hasan et al. [115] applied the presented BSMA for solving the unit commitment problem (UCP). Khelfa C et al. [120] used an SMA to improve the response time for an ambulance dispatching problem. Mehrtash Eskandaripour et al. [121] proposed a SWMM-SMA model to design a low-impact development (LID) approach for urban area management practices. To assess this model, an Iranian urban area was used as the object of the study and the analyses showed that the proposed model was more effective.

#### 5.1.3. Parameter Optimization

Many researchers use SMA variants to optimize the process parameters of equipment, management systems, and projects. Izci D et al. [30] proposed a hybrid SMA variant and evaluated it in a direct current motor and automatic voltage regulator. Izci D et al. [33] also proposed an ISMA to adjust the parameters of DC motor speed and AVR control systems. Pham Vu Hong Son et al. [108] used a hybrid AOSMA to optimize the multi-parameters of planning time, cost, quality, and safety in the construction industry. Li YiFei et al. [43] used the combined methods of polynomial chaos expansion and SMA for the multi-parameter identification of concrete dams. Pham Vu Hong Son et al. [103,113] also used a hybrid ASSMA to obtain the project’s multi-objective time, cost, quality, and environmental trade-off values. Zhang Y et al. [112] used MOSMA for the parameter optimization of laser cladding. F. Loucif et al. [122] presented a new application of SMA for the optimization of backstepping controller parameters for the tracking control and perturbations rejection of a robot manipulator. Ding P et al. [123] proposed an SMA to optimize the parameters of micro-milling, such as material removal rate, processing costs, and processing time.

### 5.2. Machine Learning

**Figure 9:**
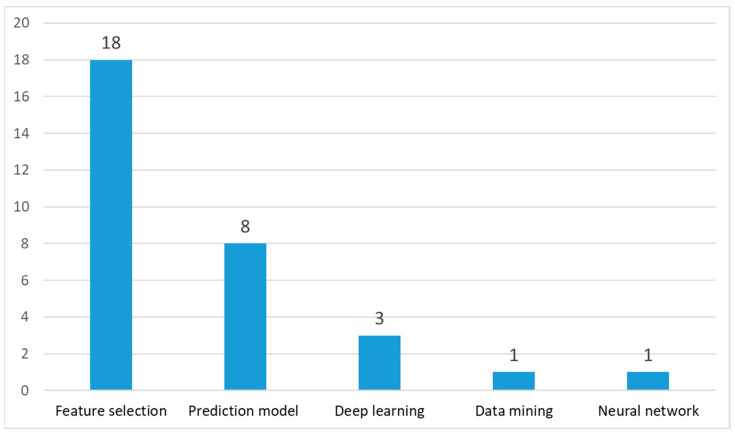
Classification and numbers of publications on machine learning between 2022 and 2023.

Machine learning appears in the second position in Figure 7. Due to its successful application for optimization problems, researchers proposed SMA and its variants for machine learning. We classified the machine learning algorithm into five sub-categories: feature selection, data mining, prediction model, neural networks, and deep learning. The details of the publications are presented in Figure 9.

#### 5.2.1. Feature Selection (FS)

Feature selections (FSs) are an effective method used to enhance the performance of machine learning algorithms by minimizing the data features’ numbers and data preprocessing steps. Many researchers have used the SMA for feature selection purposes. Hu J et al. [40] presented a binary DFSMA (called BDFSMA) and evaluated it using 12 datasets in the UCI repository. The experiment demonstrated that the BDFSMA improved the accuracy and decreased the number of features compared with other algorithms. Ramin Ghiasi et al. [54] employed a binary SMA (BSMA) for feature subset selection to improve the performance of structural damage classifications. Qiu F et al. [56] proposed a wrapper gene selection method based on BISMA and assessed it in 14 gene expression datasets. Yang P et al. [62] proposed a load identification method based on the improved slime mould algorithm–generalized regression neural network (ISMA-GRNN) and the ISMA-GRNN achieved higher accuracy and precision values for load identifications obtained from the simulation results. Qiu F et al. [69] developed a BGLSMA for the feature selection from 14 high-dimensional gene datasets and the experiments verified that the discrete BGLSMA was a promising approach for features selection purposes. Zhou X et al. [81] proposed a binary SMA (bLASMA) and evaluated it using 18 datasets of varying dimensions obtained from the UCI machine learning repository. From the experiment results, it can be observed that the bLASMA outperforms other algorithms. Ahmed A Ewees et al. [85] developed a hybrid of SMA and MPA (SMAMPA) and evaluated it using a UCI dataset and quantitative structure–activity relationship (QSAR) models. Yuheng Guo et al. [87] employed an SMA for the parameter optimization of an SVM and examined a 69-group glass chemical composition dataset to classify ancient glass products. Javidan SM et al. [89] combined SM and SVM classifiers to diagnose three apple tree diseases. Anji reddy Vaka et al. [91] proposed a hybrid WOA-SMA and applied it to BreakHis and IDC datasets to evaluate breast cancer classifications. Khan AA et al. [100] presented a hybrid of an SMA with GWO for feature selection purpose and evaluated it in UCI repository datasets by comparing it to other algorithms. Ewees AA et al. [101] also evaluated the performance of the proposed GBOSMA in several benchmark datasets to solve feature selection problems, which showed that the GBOSMA superseded the other models. H.S. Hassan et al. [114] proposed a binary SMA for feature selection purposes. Mehwish Zafar et al. [124] used an SMA to extract informative features and input them into SVM and KNN classifiers to design a multi-classification of skin lesions. Sayed GI et al. [125] introduced a pistachio species classification method on the basis of an SMA. Wei X et al. [126] proposed an SMA-VMD-WTD model to identify and eliminate transient electromagnetic signal noise.

#### 5.2.2. Prediction Model

The use of this model is a popular way of utilizing an SMA to create a prediction model to improve predication accuracy results.

Nemani R et al. [39] introduced a statistical data mining stage for intelligent rainfall predictions using an SMA and a deep learning (SDMIRPSMODL) model and evaluated it in a rainfall dataset. AlRassas AM et al. [127] used an SMA to improve the developed timeseries forecasting model for oil production predictions. Additionally, the ANFIS-SMAOLB model was evaluated using an oil production dataset. Gao H et al. [88] proposed a prediction model based on modified SMA and SVM algorithms to predict the employment stability of postgraduates. Shi B et al. [90] presented a framework where the JASMA was fused with a common kernel learning SVM for conducting an effective analysis of recurrent spontaneous abortions (RSAs). The experimental results indicated that the proposed JASMA-SVM was a promising tool when used for RSA predictions. Samantaray S et al. [97] proposed an ANFIS-PSOSMA model to predict river flood discharge (QFD) results considering the data obtained from four gauging stations in the River Brahmani, Odisha India. From the evaluation, it was observed that the proposed model had the highest accuracy. Zhang J. et al. [104] used six performance indicators, including an SMA-ANN model, to predict the settlements of single footings on soft soil reinforced by rigid inclusions, and the SMA-ANN outperformed the other models according to the experimental results. Peng C et al. [107] optimized SVR’s hyperparameters with an MOSMA, and two datasets of spindle vibration and milling data were tested for evaluating the performance of the MOSMA-SVR by comparing it with seven other prediction models. Tiachacht S et al. [128] presented an SMA-based method for structural damage detection, localization and quantification, and compared it to the MPA-based method. The results show that the proposed method can predict the location and level of damage with higher accuracy. Zhou J. et al. [129] introduced a COSMA-RF method for the cutting force prediction of conical pick cutting. 

#### 5.2.3. Deep Learning (DL)

SMA also has some advantages in optimizing deep learning frameworks. Shi B et al. [130] combined a multiple-strategy SMA (MSSMA) with a kernel extreme learning machine, called MSSMA-KELM. The MSSMA-KELM was applied to a pulmonary hypertension (PH) analysis of arterial blood gas. According to the experiments, the MSSMA-KELM is a promising technology. Lan Ngoc-Nguyen et al. [131] introduced an SMA to detect and monitor a suspension footbridge’s overall damage. The experimental results proved that the SMA was more reliable at detecting the damage location and determining the damage’s degree than the CS and GA. Hamza MA et al. [132] introduced an SMO model with a bidirectional gated recurrent unit (BiGRU) model to forecast traffic conditions in smart cities. The simulation results proved the proposed model’s superiority. 

### 5.3. Energy Optimization

**Figure 10:**
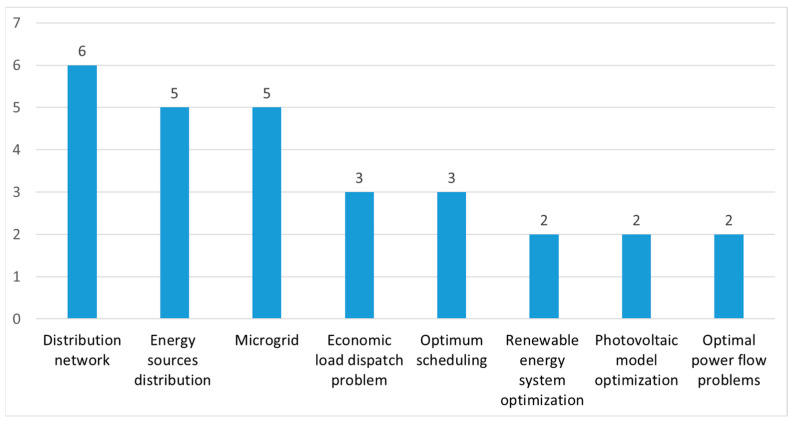
Detailed classification and numbers of the reviewed literature on energy optimization.

As shown in Figure 7, the application of energy optimization is in the third position. We further divided the energy application factor into eight sub-categories. The clarification and number of publications on energy optimization’s application between 2022 and 2023 are presented in Figure 10.

#### 5.3.1. Distribution Network

Pan JS et al. [68] proposed an MFSMA for the dynamic distribution network reconfiguration problem. Ma TX et al. [64] employed an ISMA for DC distribution network fault locations. Wang HJ et al. [133] applied the proposed PSMA to the distribution network reconfiguration (DNR) problem. Additionally, the experimental results show that the PSMA is an excellent method for solving the DNR problem. 

#### 5.3.2. Energy Sources Distribution 

Abid MS et al. [134] used a proposed CSMA to assess the optimal load shedding technique of a distribution system. Kanchan Pawani et al. [59] employed a comprehensive learning wavelet-mutated SMA to optimize a combined heat and power dispatch problem. Bhandakkar AA et al. [92] used an ISMA to optimize the layout of a hybrid power flow controller. Behera et al. [109] induced an MOISMA to solve a multi-renewable source-based energy management problem. Mohamed Zellagui et al. [135] used the SMA approach to determine the best simultaneous allocation of multiple fault current limiters (FCL) units in the standard IEEE 69-bus test system. The simulation results demonstrated the efficiency and accuracy of the SMA to reduce the fault courant in the electrical distribution system (EDS). Ahmadianfar I et al. [136] proposed an MSMA to optimize operating strategies to forecast the problem of a hydropower multiple reservoir. Wu X et al. [137] applied an SMA to optimize the allocation of water resources in Wuzhi.

#### 5.3.3. Microgrid (MG)

A Chakraborty et al. [138] proposed an SMA to minimize total operational costs via the energy management of a grid-connected low-voltage microgrid (MG), and the SAM approach was a better EM with a lower operational cost for the MG than the other methods. Behera S et al. [139] employed an SMA for the operation management of microgrids. Behera S et al. [140] also used the proposed MSMA to design an optimal battery management system in a microgrid. Liu Z et al. [141] used an SMA algorithm for the power transaction of a microgrid and grid master–slave game. Zare P et al. [142] suggested a novel control technique to perform frequency regulations in an offshore fixed-platforms microgrid system based on fractional-order hybrid controllers, where the coefficients were adjusted by the SMA.

#### 5.3.4. Economic Load Dispatch (ELD) Problem

Pawani K et al. [143] introduced a modified SMA for economic load and emission dispatch problems. Singh T et al. [53] suggested a proposed CSMA to solve an economic load dispatch (ELD) problem. Kamboj VK et al. [144] applied an SMA to solve an ELD in an electric power system.

#### 5.3.5. Optimum Scheduling

Dhawale D et al. [47] used the proposed CSMA to offer a solution to optimal generation scheduling of a vehicle to grid (V2G) operation. Bhadoria A et al. [51] proposed a hybrid CSMA-SOA for solving the generation scheduling problem of a realistic power system. Abid MS et al. [145] applied an SMA-based approach to identify the optimal charging strategies of electric vehicles (EVs). 

#### 5.3.6. Renewable Energy Systems (HRES) Optimization 

Miao H et al. [50] proposed an MSMA and applied it to a Chinese cascade hydropower reservoir system to optimize the annual power generation. Olalekan Kunle Ajiboye et al. [146] employed an SMA for the optimization of hybrid renewable energy systems (HRESs).

#### 5.3.7. Photovoltaic Model Optimization

Lin H et al. [57] developed an ASMA and employed it to attain the optimal parameters of PV models. MS K [117] proposed a discrete time-based SMA to provide effective support to a buck converter-based maximum power point tracking (MPPT) controller for solar photovoltaic (SPV) systems.

#### 5.3.8. Optimal Power Flow (OPF) Problem

Al-Kaabi M et al. [78] employed an MOSMA to solve multi-objective optimal power flow problems. Farhat M et al. [147] used the proposed ESMA for solving the optimal power flow (OPF) problem. 

### 5.4. Image Segmentation

Image segmentation separates an image into sub-regions or objects depending on its components; it is a useful method for various applications, such as multi-threshold image segmentation, medical image processing, sonar image recognition, and image compression.

The multi-threshold image segmentation method is one frequently used by scholars in the domain of image processing. Dipak Kumar Patra et al. [34] proposed an SMA-based multilevel thresholding technique to perform breast DCE-MRI segmentations which was evaluated in 200 DCE-MRI images of 40 patients. The simulation results indicate the proposed method outperforms the other compared methods. Chen X et al. [96] proposed an improved SMA (ASMA) for the multilevel image segmentation (MLTIS) of a lupus nephritis (LN) diagnosis, called ASMA-based MLTIS. The proposed approach was assessed and proved to be an efficient image segmentation method for the LN images. Mehbodniya A et al. [148] used an SMA to attain optimal threshold values for host images and the experiments demonstrated the proposed approach was better than the others. Yuanyuan Jiang et al. [149] presented an improved ISMA for a multi-level thresholding image segmentation and symmetric cross-entropy processes to perform image segmentation tasks. Shi M et al. [150] proposed an RWGSMA for multi-threshold image segmentation purposes. To evaluate the RWGSMA’s efficiency, instances of lupus nephritis were used and the experiments demonstrated the method’s superiority. 

In addition to the multi-threshold image segmentation, the researchers used image segmentation technology to assist in performing the medical diagnoses [34,96,150]. Krishna Gopal Dhal et al. [35] used an ISMA to perform illumination-free white blood cell (WBC) segmentations.

Other image segmentation applications also exist in the literature. Yutong G et al. [79] designed a DCNN–ELM–FSMA model, where a fuzzy SMA (FSMA) was applied. The DCNN–ELM–FSMA’s performance was evaluated in three sonar datasets and the results indicated that the proposed mode was more accurate in its sonar image classifications. Ren L et al. [84] developed an enhanced version of an SMA called MGSMA and applied it to a multi-level image segmentation model. The MGSMA-based MLIS method was compared to eight other models at both high-and low-threshold levels using 10 images obtained from BSDS500, and the results revealed that the suggested approach could deliver high-quality image segmentation results. Chavan PP et al. [98] suggested an image compression technique to perform a vector quantization (VQ) with the K-means Linde–Buzo–Gary (KLBG) model. In the encoding stage, the hybrid GA and SMA was employed for generating an optimal codebook. The comparation results revealed the proposed method performed better than the others. Debnath A et al. [151] presented an improved image-denoising technique that was based on the combination of cascaded filters. An SMA was used to combine different filters. From the visual and quantitative analyses, the proposed technique improved the quality of the images. 

### 5.5. Scheduling Optimization

There are various SMA variants applied for scheduling optimization techniques, including path finding, autonomous mobile robots, JSSP, and others.

Path planning is the location of an optimal route from a starting point to a targeted point under certain contains and is widely applied in various domains, such as autonomous mobile robot (AMR) logistics and safety evacuations. Yang H et al. [61] proposed an improved SMA to locate the optimal path for unmanned equipment in fire rescue missions. Ling Zheng et al. [66] proposed a Lévy flight–rotation SMA (LRSMA) for the path planning of an autonomous mobile robot (AMR). The experiments showed that the proposed approach could identify the optimal path without obstructions and with higher accuracy and stability outcomes. Hu G et al. [152] proposed an HG-SMA to build a path planning model on the basis of a Said Ball curve. The HG-SMA approach was evaluated in three workplaces with rectangular, circular, and mixed obstacles, and the results revealed that the suggested algorithm achieved better values for the path distance, smoothness, and stability. Yueming Q et al. [153] developed both single/multi-objective methods for the path planning of automatic guided vehicles (AGVs) based on the SMA.

The motion control of autonomous mobile robots was also optimized with SMA and its variants. Yin S et al. [76] proposed both single- and multi-objective versions of an EOSMA and applied them to solve the inverse kinematics (IKs) of manipulators. The test indicated the proposed EOSMA had a shorter computation time than the other algorithms. Li X et al. [93] developed a hybrid algorithm of the SMA with WOS (SMWOA) for the joint trajectory planning of a robot. A 6-DOF UR5 manipulator was used to evaluate the SMWOA’s efficiency and the results showed that the running time of the joints was shorter.

Job shop scheduling problems play an important role in the production process and many researchers have applied the SMA and its variants to JSSPs. Dan Li et al. [116] introduced a multi-strategy SMA named GCSMA to the flexible job shop scheduling problem (FJSP).

In addition, Zheyuan Wang et al. [154] designed an SMA-based energy-efficient traffic scheduling method (SMA-ETSM) for a software-defined network (SDN).

### 5.6. Network 

Several experiments joined SMAs and variants for a wireless sensor network (WSN). 

Yuanye We et al. [31] proposed an SSMA for solving the WSN coverage problem. According to 13 groups of WSN coverage optimization experiments, the SSMA outperformed the other algorithms regarding the network node energy, the service quality, and network survival time. J Sengathir et al. [37] proposed an adaptive opposition learning–improved SMA (AOLISMA)-based optimization routing technique to ensure reliable data dissemination among UAVs, extend network lifetime, and minimize energy consumption levels. The experimental results demonstrate that the proposed AOLISMA can enhance the throughput and reduce the control overhead. Alwan MH et al. [155] integrated an SMA into an intrusion detection system (IDS) in wireless sensor networks for anomaly detection purposes. The SMA was used to decrease the number of features. From the evaluation of the NSL-KDD dataset, it was observed that the proposed method significantly improved the value.

### 5.7. Others

In this section, we categorized the SMA variants that only used the benchmark functions or were not applicable to the classification criterion described above as “other”.

Using the benchmark or basic functions to evaluate SMA variants are common methods used by researchers in the field. Xuebin Cai et al. [45,156] introduced a general multi-objective SMA and compared it with seven advanced multi-objective algorithms on 28 basis functions, which demonstrated that the proposed MSMA outperformed the other algorithms. Alfadhli J et al. [157] proposed an adaptive fluctuant population size SMA (FP-SMA) and evaluated it in 13 standard and 30 IEEE CEC2014 benchmark functions. Bujok P et al. [158] compared 17 variants of the SMA algorithm with 16 other optimizers on CEC 2011. The result revealed that most of the new SMA variants performed better than the original SMA.

## 6. Discussion 

As previously discussed, the SMA is a new metaheuristic algorithm and researchers have developed numerous SMA variants by adding strategies or hybridizing additional algorithms for various application domains. The advantages and disadvantages of SMAs are summarized in Table 2.

### 6.1. Advantages of the SMA

Firstly, the SMA has less parameters to set and a simpler structure compared to other algorithms. As described in Section 3, there were only three parameters ($\vec{W}$,$\vec{vb}$,$\vec{vc}$) that were adjusted adaptively with the number of iterations, and the hyperparameter *z* was empirically pre-set to 0.03. The SMA pseudo-code indicated that there were only double loops and Equation (1) represents the exploration.

Secondly, the SMA has excellent scalability which was proved by various SMA variants based on the hybridization with other algorithms and adding strategies conducted in this study. 

As a result of the simple structure and good scalability previously mentioned, the SMA demonstrated its superiority when solving optimization problems. Additionally, the popularity of the SMA was also indicated in recent publications.

Since SMAs have a multi-point search ability, which is conducted by position and negative feedback weights $\vec{W}$ presented in Equation (4), the SMA can rapidly obtain the optimal candidate solution from the neighbor search region. Therefore, the SMA has a strong exploitation capability.

According to the wall-clock time costs of the SMA and other algorithms, the computational time of the SMA has a considerable advantage over other algorithms [19].

Finally, the SMA uses positive and negative feedback weights $\vec{W}$, presented in Equation (4), to regulate an individual’s position. The weight is also calculated according to the environment of the search individual. When high-quality food is present, the weight near the location increases; otherwise, the weight reduces, causing the individual to explore another location. The adaptive mechanism enables the SMA to adjust the search pattern according to the change in the environment and location of the individuals, thus stepping out of local stagnation. The vibration parameter $\vec{vb}$, which fluctuates randomly between [−*a*, *a*] and finally approaches zero, ensures a rapid convergent rate.

### 6.2. Disadvantages of the SMA

The SMA has gained significant popularity in solving a diverse range of problems since its proposal. However, it presents some limitations and drawbacks.

The main problems of the SMA are the local optimal stagnation, low convergent speed, and low accuracy while handling multimodal and high-dimensional problems, similar to many metaheuristic optimization algorithms. On increasing the problem size, algorithms’ difficulties in finding the solution also grow. Therefore, the algorithm would find it difficult to solve multimodal, high-dimensional, and nonlinear problems, even if they achieve satisfactory results for unimodal difficulties. The basic version of the SMA was designed to solve continuous single-objective optimization problems. It is inevitable for the SMA to have problems of falling local optimum, slow convergent speed, and low accuracy in the face of high-dimensional and multimodal optimization problems.

Insufficient global exploration is another drawback of the SMA. There are two reasons for this. One is that the initial population of the SMA is generated randomly, resulting in an unstable and poor population quality. The poor diversity of the population affects and reduces the search scope and convergent speed as the problem dimension increases and the search space expands. The other reason is due to the multi-point local search mechanism of the SMA. This mechanism can greatly improve the exploitation ability of the SMA. However, the strong local search capability is achieved at the cost of exploration, resulting in an imbalance in the exploitation and exploration capabilities.

Finally, according to the review of publications on SMAs between 2022 and 2023, we observed that there was a small portion of multi-objective and discrete versions of SMA variants (see Figure 5 and Figure 6). This may be a future research direction for researchers studying SMAs.

## 7. Conclusions and Future Work

This paper presented a detailed review of the SMA, demonstrating that it is a reliable algorithm for solving numerous optimization problems. This paper also offers theoretical and practical information and analysis to researchers who wish to improve their work based on SMAs.

This review paper presented the theory of SMA and the recent development of SMA variants. First of all, we introduced the concept and theory of the SMA, including the mathematical model, pseudo-code, and flowchart. Then, we collected 130 papers from various well-known publishers on the SMA between 2020 and 2023. Based on this collection of 130 papers, we classified the SMA variants by the methods they employed to improve the performances of strategy embedding and the hybridization of both algorithms. From an analysis of the review, we observed that the number of the multi-objective and discrete versions of SMA variants was less than the compared single-objective and continuous versions. Furthermore, the application of the SMA and its variants were categorized into seven domains: engineering optimization, energy optimization, machine learning, network, scheduling optimization, image segmentation, and others. The wide scope of applications proved that the SMA is a promising algorithm used for solving various real-world optimization problems, especially in the fields of engineering and energy. Finally, we discussed the advantages and disadvantages of using the SMA. The advantages of the SMA include the following: few parameters to set, simple structure, excellent scalability, strong exploitation capability, reduced computational time, superiority for optimization problem, and adaptive and vibration parameters. In contrast to the advantages of the SMA, they had several shortcomings. They presented the same problems of easy trapping in local optima, slow convergent speed, and low accuracy when handling high-dimensional and multimodal problems, which other metaheuristic algorithms commonly experienced. The SMA also presented the disadvantages of an insufficient global search capability, an imbalance between exploration and exploitation capabilities, and few multi-objectives and discrete SMA variants. 

Although significant results concerning SMA research have been achieved, there is still some room for the improvement of the SMA for future works. Firstly, the multi-objective and discrete SMA variants are worthy of further research. As shown in Figure 5 and Figure 6, only 22 papers concerning the multi-objective version and 11 papers concerning the discrete version exist in the literature, compared to 106 papers on the single-objective version and 117 papers on the continuous version. Secondly, the methods adopted for the SMA and its variants can be extended to neural networks and extreme learning machining. Finally, it is a promising direction in the research to apply the SMA and its variants to solve real, complex, dynamic, and large-scale engineering optimization problems.

## Figures and Tables

**Table 1:** Top 3 countries ranked by the number of SMA publications since 2022.

Country	Rank	Number of Publications
China	1	56
India	2	30
Egypt	3	6
Iran	3	6

**Table 2:** Advantages and disadvantages of SMAs.

Advantages	Disadvantages
- Few parameters to set and simple structure - Excellent scalability - Superiority for optimization problem - Strong exploitation capability - Reduced computational time - Adaptive and vibration parameters	- Easily trapped in local optimum, low convergent rate, and low accuracy in the face of high-dimensional and multimodal problems - Insufficient global search capability - Imbalance between exploration and exploitation capabilities - Few multi-objectives and discrete SMA variants

## Data Availability

No new data available for this review.

## Appendix A

**Table A1:** Summary of SMA Variants with Different Strategies.

No	Strategies	References
1	Opposition-based learning	Sharma et al. [25], Izci D et al. [30,33], Lin H et al. [57], Son, P. V. H. et al. [108], Dipak Kumar Patra1 et al. [34], Krishna Gopal Dhal et al. [35], Liang Xu et al. [36], Sengathir J. et al. [37], Houssein EH et al. [82], AlRassas AM et al. [127], Pawani K et al. [143]
2	Chaotic strategy	Rizk-Allah RM et al. [42], Li Yi Fei et al. [43], Yin Shihong et al. [44], Xuebing Cai et al. [45], Yuan L et al. [46], Dhawale D et al. [47], Chen H et al. [48], Zhong C et al. [49], Abid MS et al. [134], Miao H C et al. [50], Bhadoria A et al. [51], Sarhan S et al. [52], Singh T [53]
3	Mutation operator	Lin H et al. [57], Ramin Ghiasi et al. [54], Deng L et al. [55], Qiu F et al. [56], Yin S et al. [58], Pawani et al. [59], Zheng R et al. [60], H. Yang et al. [61], Yang P et al. [62]
4	Lévy flight	Ling Zheng et al. [66], He W et al. [67], Pan JS et al. [68], Qi A et al. [63], Qiu F et al. [69], Jui JJ et al. [70], Kundu T et al. [71]
5	Crossover operator	Rizk-Allah RM et al. [42], Ramin Ghiasi et al. [54], Qiu F et al. [56], Qi A et al. [63], Ma TX et al. [64]
6	Elite strategy	Yuan L et al. [46], Miao H C et al. [50], Sarhan S et al. [52], Kaveh A et al. [72], Luo Qifang et al. [73]
7	Greedy selection	Liu J et al. [70], Shubiao Wu et al. [75], Yin S et al. [76]
8	Fuzzy	Prabhu, M et al. [77], Al-Kaabi M et al. [78], Yutong G et al. [79]
9	Neighborhood search	Yuanfei Wei et al. [80], Zhou X et al. [81]
10	Sigmoid function	He W et al. [67], Örnek BN et al. [83]
11	Gaussian strategy	Shubiao Wu et al. [75], Ren L et al. [84]

## Appendix B

**Table A2:** Summary of SMA Variants Hybridized with Other Algorithms.

No	Hybrid Algorithms	References
1	Equilibrium optimizer (EO)	Yin S et al. [58], Yin S et al. [76], Yuanfei Wei et al. [80], Luo Qifang et al. [73]
2	Differential evolution (DE)	Krishna Gopal Dhal et al. [35], Chen H et al. [48], Qiu F et al. [56], Shubiao Wu et al. [75]
3	Support vector machine (SVM)	Yuheng Guo et al. [87], Gao H et al. [88], Javidan SM et al. [89], Shi B et al. [90]
4	Whale optimization algorithm (WOA)	Anji Reddy Vaka et al. [91], Bhandakkar AA et al. [92], Li X et al. [93]
5	Simulated annealing (SA) algorithm	Izci D et al. [30], Leela Kumari Ch et al. [94]
6	Teaching–learning-based optimization (TLBO)	Zhong C et al. [49], Kundu T et al. [71]
7	Seagull optimization algorithm (SOA)	Bhadoria A et al. [51],Das G et al. [95]
8	Artificial bee colony (ABC)	Ma TX et al. [64], Chen X et al. [96]
9	Sine cosine algorithm (SCA)	Örnek BN et al. [83]
10	Marine predators algorithm (MPA)	Ewees A.A. et al. [85]
11	Particle swarm optimization (PSO)	Samantaray S et al. [97]
12	Genetic algorithm (GA)	P.P. Chavan et al. [98]
13	Evolutionary algorithm (EA)	Chauhan S et al. [99]
14	Gray wolf optimization algorithm (GWOA)	Khan AA et al. [100]
15	Gradient-based optimizer (GO)	Ewees A.A.et al. [101]
16	Quadratic approximation (QA)	Chakraborty P et al. [102]
17	Tournament selection (TS)	Son PV et al. [103]
18	Artificial neural network (ANN)	Zhang J et al. [104]
19	Moth-flame optimization algorithm (MFOA)	Hussein SN et al. [105]
20	Pattern search algorithm (PSA)	Bala Krishna A et al. [106]
21	Support vector regression (SVR)	Peng C et al. [107]
